# Oxidative Stress Following Intracerebral Hemorrhage: From Molecular Mechanisms to Therapeutic Targets

**DOI:** 10.3389/fimmu.2022.847246

**Published:** 2022-03-09

**Authors:** Yan Zhang, Suliman Khan, Yang Liu, Guofeng Wu, V. Wee Yong, Mengzhou Xue

**Affiliations:** ^1^ Department of Cerebrovascular Diseases, The Second Affiliated Hospital of Zhengzhou University, Zhengzhou, China; ^2^ Academy of Medical Science, Zhengzhou University, Zhengzhou, China; ^3^ Department of Emergency, Affiliated Hospital of Guizhou Medical University, Guiyang, China; ^4^ Hotchkiss Brain Institute and Department of Clinical Neurosciences, University of Calgary, Calgary, AB, Canada

**Keywords:** intracerebral hemorrhage, reactive oxygen species, oxidative stress, anti-oxidative stress, brain injury

## Abstract

Intracerebral hemorrhage (ICH) is a highly fatal disease with mortality rate of approximately 50%. Oxidative stress (OS) is a prominent cause of brain injury in ICH. Important sources of reactive oxygen species after hemorrhage are mitochondria dysfunction, degradated products of erythrocytes, excitotoxic glutamate, activated microglia and infiltrated neutrophils. OS harms the central nervous system after ICH mainly through impacting inflammation, killing brain cells and exacerbating damage of the blood brain barrier. This review discusses the sources and the possible molecular mechanisms of OS in producing brain injury in ICH, and anti-OS strategies to ameliorate the devastation of ICH.

## 1 Introduction

Intracerebral hemorrhage (ICH) is a leading health problem with high mortality and adverse outcomes (1–3). The poor prognosis is attributed to the primary brain injury caused by the hematoma (4), and secondary brain injury attributed to neuroinflammation, excitatory amino acid, oxidative stress (OS), cytotoxicity of blood, hypermetabolism and spreading depression (5–7). In addition to limited progress in the management of ICH patients, effective treatment options are yet to be developed for the secondary brain injury of ICH (8–12).

OS refers to the overbalance of reactive oxygen free radicals and/or deficiency of antioxidant systems in cells. Reactive free radicals include reactive oxygen species (ROS) and reactive nitrogen radicals (RNS) (13). RNS mainly consists of nitric oxide and its derivatives. ROS are molecules composed of oxygen free radicals (eg superoxide anion radical (O2·−), hydroxyl radical (·OH)) and nonradical oxidants (for example hydrogen peroxide (H_2_O_2_) and singlet oxygen (^1^O_2_)) (14). ROS are difficult to detect directly due to its highly reactive state with a short half-life; therefore, the oxidized products of DNA, protein, and lipid are documented as indirect readouts of the levels of OS (15).

The brain is an organ that is particularly vulnerable to OS damage because it is rich in lipids and iron, but is relatively deficient in antioxidants such as nuclear factor erythroid-2 related factor 2 (Nrf2), glutathione peroxidases, heme oxygenase, and superoxide dismutase (16). Considerable evidence implicate OS in the pathophysiology of many different brain diseases, including neurodegenerative disorders, depression and ischemic stroke (17, 18). Most importantly, OS is also a primary mediator that results in secondary brain injury after ICH (19). Accumulating evidence have revealed that the level of ROS is increased accompanying antioxidant enzyme reduction in the brain after ICH (19, 20). Anti-OS strategies improve neurological function, and attenuates inflammatory responses and blood brain barrier (BBB) damage caused by OS after ICH (21). This review seeks to illustrate the knowledge of ICH-related OS, so as to support the theoretical basis for intervention against OS to improve the prognosis of this devastating stroke.

## 2 ICH Leads to OS

The Keap1/Nrf2/ARE pathway is activated by ROS to resist OS damage after ICH as an adaptive defense mechanism. Unfortunately, cell death is inevitable due to excessive OS (22). Increasing evidence indicate that OS is triggered after ICH and is a critical factor for ICH-induced secondary brain injury (23). Oxidation of protein in the perihematomal region was observed to be increased by approximately 2.1-fold in collagenase-injected rats, as compared with contralateral striatum (24). ROS, 8-OHDG, 3-nitrotyrosine and malondialdehyde levels were increased in a rat ICH model (25). Similarly, ICH also led to an increase in the levels of carbonylated and nitrosylated proteins at 12 hours after onset (26). Moreover, hydroethidine signals were enhanced in parallel with the elevated level of matrix metalloproteinase-9 (27). These results indicate that the redox environment is changed after ICH and severe OS damage occurs in the perihematoma brain tissues.

## 3 The Source of ROS/RNS Following ICH

**Figure 1:**
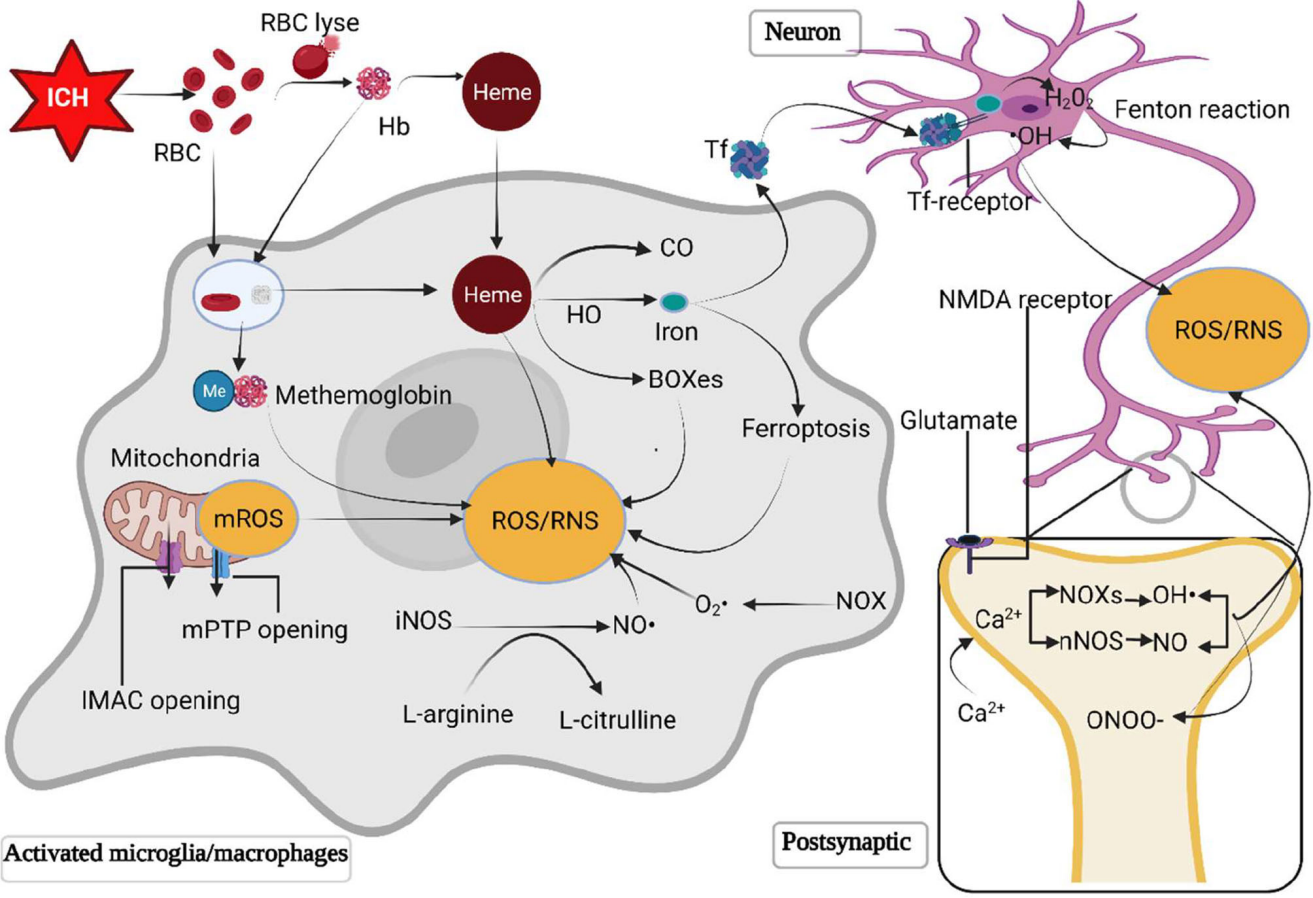
The primary sources of ROS/RNS following ICH. Following ICH, the destruction of erythrocytes in a hematoma releases hemoglobin and heme. The RBC and degraded hemoglobin and heme are phagocyted by microglia/macrophages. Hemoglobin is then oxidized to methemoglobin to generate free radicals. Heme is metabolized by heme oxygenase into biliverdin, iron and carbonic oxide. Iron is transferred to neurons mediated by the transferrin delivery system, where iron reacts with H_2_O_2_ through the Fenton reaction to produce •OH, which is more toxic. The opening of inner membrane anion channels and mitochondrial permeability transition pore results in ROS release. Besides, excessive glutamate following ICH activates postsynaptic neuron NMDA receptors, promotes Ca^2+^ influx and intracellular Ca^2+^ overload, triggering the synthesis of neuronal nitric oxide and superoxide *via* activating neuronal nitric oxide synthase and nicotinamide adenine dinucleotide phosphate oxidases (NOXs) respectively. The polymerization of nitric oxide and superoxide forms peroxynitrite. Moreover, NOX in microglia/macrophages is activated to generate O_2_· and NOS converts L-arginine to L-citrulline to produces NO. HO, heme oxygenase; Hb, hemoglobin; H_2_O_2_, hydrogen peroxide; •OH, hydroxyl radical; ICH, intracerebral hemorrhage; IMAC, inner membrane anion channel; mPTP, mitochondrial permeability transition pore; NOXs, nicotinamide adenine dinucleotide phosphate oxidases; NO, nitric oxide; NOS, nitric oxide synthase; ONOO, peroxynitrite; RBC, red blood cell; ROS/RNS, reactive oxygen/nitrogen species; O_2_·, superoxide anion radical; Tf, transferrin.

ROS/RNS can be produced by several pathological processes after ICH, including mitochondria dysfunction, metabolic products of red blood cell, excessive activation of N-methyl-D-aspartic acid (NMDA) receptors through glutamate, and inflammatory cells (**Figure 1**).

### 3.1 Mitochondria Dysfunction

The complex I and III of mitochondrial respiratory chain are not only the main source of ROS production but also the primary target organelles of ROS damage (28). One study found that mitochondrial oxygen consumption was decreased approximately 60% in ICH patients (29). Another study reported that the expression levels of mitochondrial complex I were significantly reduced in the ICH group (30), indicating mitochondrial dysfunction. The opening of inner membrane anion channel and the mitochondrial permeability transition pore results in the change of intracellular and intramitochondrial oxidation environment, which triggers ROS release. High levels of ROS combined with sustained activated mitochondrial permeability transition pore lead to a ROS burst, which exacerbates mitochondrial damage. This destructive effect can propagate from one mitochondrion to another (23). OS damage associated with ICH was alleviated by inhibiting the activation of mitochondrial permeability transition pore and countering the overproduction of mitochondrial ROS (mROS) (31).

### 3.2 Metabolic Products of Hemoglobin

Following ICH, red blood cells degrade and release hemoglobin. The latter results in hemin (Fe^3+^ protoporphyrin IX) (32), which is gradually phagocytosed by resident microglia, invading monocyte-derived macrophages, astrocytes and neurons. Following this, hemin degradation by heme oxygenase mediated cleavage of the porphyrin ring results in biliverdin and carbonic oxide, and iron is released. Biliverdin is then metabolized to bilirubin by biliverdin reductase. The free iron ions are stored in ferritin, which is degraded in lysozymes to the redox-inert hemosiderin (32).

#### 3.2.1 Hemoglobin

Hemoglobin, the primary component released by erythrocytes, is a potent mediator of OS-induced injury. Katsu et al. found that intracerebral injection of hemoglobin into rat striatum induced hydroethidine signals, which was found colocalized within vessel walls by assessing situ gelatinolytic activity (27). Yan et al. reported that injection of hemoglobin into striatum dramatically boosted the level of carbonylation and formation of malondialdehyde compared with that in the contralateral striatum at 72 hours (33). Prominently, hemoglobin oxidative injury is caused by the formation of free radicals (mainly through Fenton-type mechanism) (7), while deoxyhemoglobin spontaneously and nonenzymatically oxidizes to methemoglobin (34).

#### 3.2.2 Hemin

Hemin, the degradation substance of hemoglobin, is highly cytotoxic due to the large amount of ROS it can produce (35). *In vivo* or *in vitro*, ROS production can be significantly elevated by treatment with hemin in rat brain or in cultures of primary cortical neuron (36) and cortical astrocytes (35). The mechanism of hemin-related oxidative damage is thought to be due to its breakdown to iron by heme oxygenase (13). Indeed, hemin can directly induce the production of free radicals and damage intracellular structures (37). Hemin is a lipophilic substance that promotes lipid peroxidation by inserting into lipid membranes (38).

#### 3.2.3 Iron

Iron is a product of the degradation of hemoglobin/hemin. In a collagenase ICH model, iron was found to deposit around the hematoma 1 day after ICH, peaked at 7 days, and remained at a higher level at 14 days (39). OS induced by ferrous iron leads to DNA injury and brain damage after ICH. The mechanism of iron overload leading to ROS production after ICH mainly includes neuronal toxicity of iron *via* transferrin receptor transport system (40), and ferroptosis, an iron-dependent lipid peroxidation injury (41). Recently, ferroptosis was documented to occur in a mouse model of ICH (42). Ferrostatin-1, a specific inhibitor of ferroptosis, exerts neuroprotective effects by inhibiting lipid peroxidation, prostaglandin-endoperoxide synthase-2 and cyclooxygenase-2 (42). The iron chelator, deferiprone and clioquinol, decreased iron levels and ROS production, improved neurological outcomes and attenuated brain edema (43). Consistent with these results, Takehiko et al. reported neurological dysfunction and brain edema, and the high expression of 8-Hydroxy-2-deoxyguanosine in ICH was significantly alleviated after deferoxamine treatment (44). These observations indicate that deferoxamine and other iron chelators may be neuroprotective through reduction of OS triggered by iron.

#### 3.2.4 Bilirubin Oxidation Products

Bilirubin is the end product of hemin catabolism. Low level of bilirubin induces OS, neuroinflammation, and cell death (45). Besides hemoglobin and hemin, bilirubin oxidation products are also considered as a source of ROS after ICH. OS damage caused by bilirubin oxidation products *via* hemoglobin and Fenton reaction following ICH was first reported in 2008. Bilirubin oxidation products and malondialdehyde were significantly increased in brain tissues of the porcine ICH model, suggesting a strong oxidative stress environment (46). However, there have been no thorough information regarding bilirubin oxidation products in ICH since then.

### 3.3 Excitatory Amino Acids

Accumulating research indicates that glutamate excitotoxicity is involved in the secondary brain injury of ICH (47). After ICH, glutamate release is increased but uptake is reduced, so that the excessive glutamate continuously activates NMDA receptors in postsynaptic neurons; this promotes Ca^2+^ influx and intracellular Ca^2+^ overload, triggering the synthesis of neuronal nitric oxide and superoxide *via* activating neuronal nitric oxide synthase and nicotinamide adenine dinucleotide phosphate oxidases (NOXs) respectively (48). The polymerization of nitric oxide and superoxide can form peroxynitrite. All the three compounds, alone or in combination, can cause DNA damage (49).

### 3.4 Inflammatory Cells

Inflammation contributes to secondary brain damage after ICH (50), and microglia and infiltrated neutrophils play a key role. In addition to their release of pro-inflammatory proteases, cytokines and chemokines, microglia and neutrophils also supply excessive ROS and nitric oxide (51) in part through dysregulation of the respiratory chain. Meanwhile, a large amount of superoxide dismutase is consumed to remove free radicals, and this eventually leads to lipid peroxidation (52).

The reaction of microglia after ICH is complex. Microglia is activated by hemoglobin, iron and hemin; they attempt to degrade hemin and clear the hematoma by the microsomal heme oxygenase system (22, 53), but they also produce potentially toxic nitric oxide (22). *In vitro*, microglia exposed to erythrocyte lysis elevate ROS production (54), they also polarize from a pro-inflammatory to regulatory phenotype, and they may inhibit neuronal ROS overproduction and decrease neuronal death in a trans-well co-culture system (55). Microglia activation up-regulates the gene expression of inducible nitric oxide synthase, COX2, tumor necrosis factor, and interleukin 1β through multiple signaling pathways, which are inherently associated with OS (56). Iron and hemin digested in microglia induce ROS production and accentuate ROS synthesis from activated microglia (53).

After ICH, neutrophils from peripheral blood infiltrate into brain tissue. They directly participate in the pathological injury process of ICH by releasing a large amount of pro-inflammatory proteases, producing ROS, and damaging the BBB (57). Recently, evidence from clinical studies report that patients with ICH had higher (compared to those of patients with ischemic strokes) levels of spontaneous and induced synthesis of secondary ROS but with a decrease in metabolic resources for their synthesis (58). In animals, decreasing neutrophil infiltration by knockdown of nucleotide-binding domain leucine-rich repeat (NLR) family pyrin domain containing 3 (NLRP3) decreased myeloperoxidase levels at 24 hours following ICH (31). Neutrophil activation is accompanied by the production of large amounts of ROS; besides, nitric oxide is generated by the conversion of the amino acid L-arginine to L-citrulline *via* NOS in neutrophils (59).

## 4 The Destructive Role of OS to the Brain Following ICH

**Figure 2:**
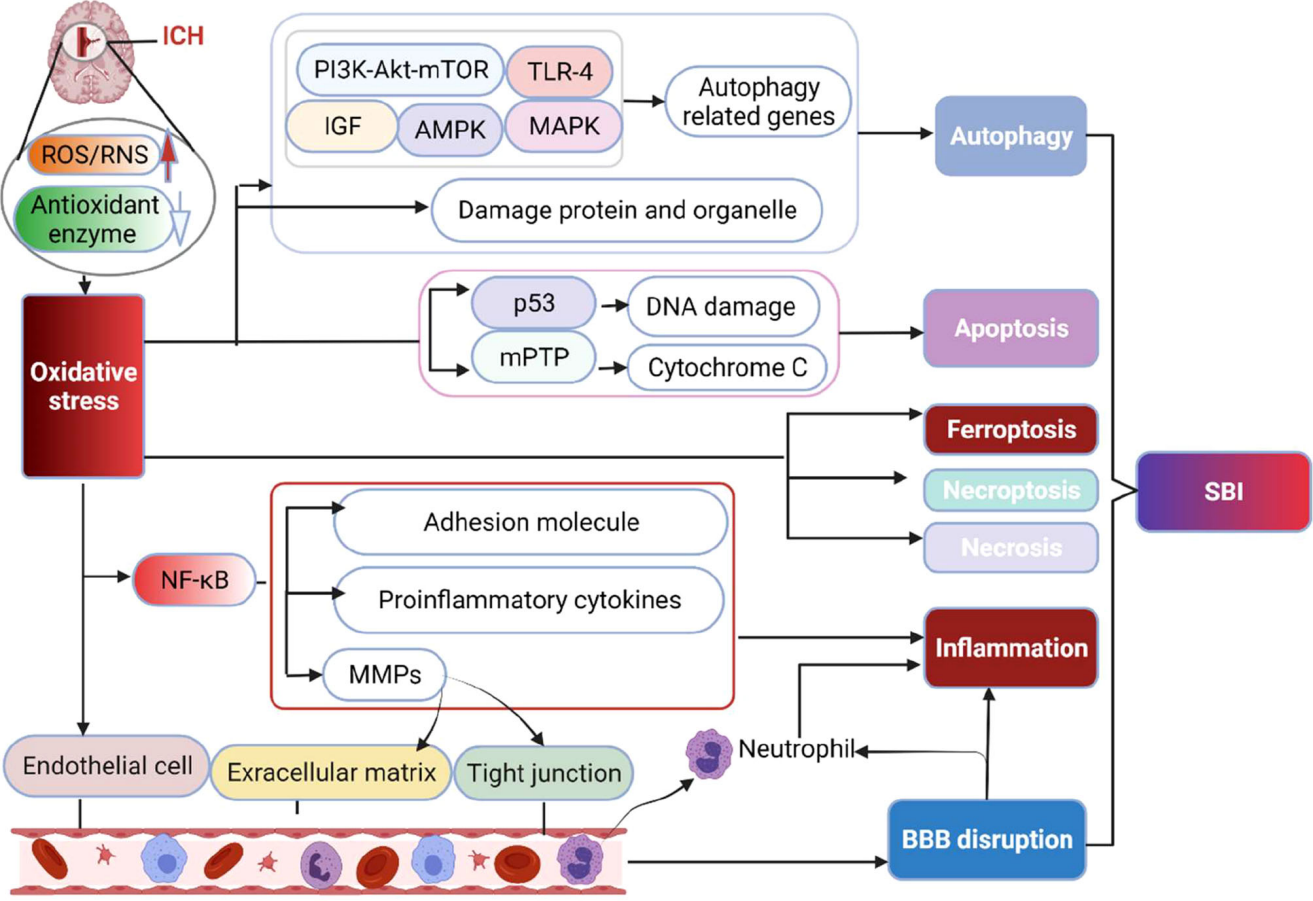
The main destructive effect of OS to brain following ICH. The production of ROS/RNS significantly exceeds the body’s antioxidant capacity after ICH, resulting in irreversible damage to lipids, proteins, and DNA, ultimately inducing multiple forms of cell death pathways (apoptosis, necrosis, necroptosis, ferroptosis and autophagy) *via* activating related molecular networks or genes. Besides, OS increases the level of NF-κB, which upregulates proinflammatory cytokines, inflammatory molecules and MMPs to result in inflammation. MMPs increase the permeability of capillaries by degrading the basement membrane and tight junction structure of cerebrovascular endothelial cells, thereby contributing to inflammation and BBB disruption. Moreover, OS can directly injure endothelial cells and damage the BBB. BBB, blood brain barrier; MMPs, matrix metalloproteinases; mPTP, mitochondrial permeability transition pore; NF-κB, nuclear factor-kappa B; SBI, secondary brain injury.

OS may destroy brain tissue *via* propagating inflammatory responses, mediating cell death, and destroying neurovascular units (**Figure 2**).

### 4.1 The Interaction Between OS and Inflammation Following ICH

Inflammation is a major component of brain injury in ICH (60). It is mediated primarily by activated microglia, infiltrating leukocytes, and pro-inflammatory molecules (eg cytokines, chemokines, and ROS) from these cells (61).

Previous investigations have demonstrated that OS promotes the release of pro-inflammatory cytokines. Correspondingly, inflammatory stimuli induce the release of antioxidant gene peroxiredoxin 2 (62). OS elaborated by polymorphonuclear neutrophils results in the opening of tight junctions of vascular endothelial cells and facilitates inflammatory cells to cross the BBB into brain tissue (63). Levels of matrix metalloproteinases can be elevated by release from activated microglia to result in increased permeability of capillaries. In this regard, matrix metalloproteinases degrade the basal lamina and disrupt the tight junctions of endothelial cells, thereby contributing to brain edema, necrosis, and BBB disruption (61). Reduction in OS by superoxide dismutase1 overexpression reduced matrix metalloproteinase-9 levels, and subsequent apoptosis in hemoglobin induced ICH injury model (27). In addition, blocking overproduction of mitochondrial ROS through a selective mitochondrial ROS antioxidant MitoQ reduces the NLRP3 inflammasome activation in FeCl_2_-treated microglia (64). Moreover, inhibiting microglial inflammation through sinomenine attenuates ROS production in ICH-challenged BV2 microglial cells (54). Thrombin is another stroke-related inflammatory mediator that has been implicated in ICH pathology. The elevated ROS and cellular apoptosis were suppressed by the ROS inhibitor N-acetyl-l-cysteine after microglia were treated with thrombin (65).

### 4.2 A Vicious Cycle of OS and Glutamate Exitotoxicity in ICH

After ICH, excessive glutamate exacerbates injury by stimulating NMDA receptors (49). Correspondingly, oxidation products were shown to enhance glutamate release through increasing connexins and pannexins based channel activity following ICH (10). ROS/RNS regulate the change of connexin and pannexin-based channel properties both in systemic vasculature and brain cells (66). Glutamate released *via* astroglial connexin 43 hemichannels mediates neuronal death through activation of pannexin 1 hemichannels (67). Current evidence indicate that pannexin 1 channels can be activated by NMDA receptors *via* Src family kinases (68). These data suggest a vicious cycle of glutamate being an important source of ROS/RNS, and OS in turn promoting glutamate release through connexins and pannexins based channels after ICH.

### 4.3 The Interplay Between OS and Brain Cell Death After ICH

The production of ROS/RNS significantly exceeds the body’s antioxidant capacity after ICH, resulting in irreversible damage to lipids, proteins, and DNA, ultimately inducing multiple cell death pathways (2, 69, 70).

#### 4.3.1 OS Initiates Brain Cell Apoptosis After ICH

Apoptosis is caspase dependent programmed cell death (71). Han and colleagues reported that hydroxyl radicals and malondialdehyde dramatically increased in an ICH animal model; terminal deoxynucleotidyl transferase deoxyuridine triphosphate nick-end labeling (TUNEL)-positive cells appeared at 6 hours, peaked at 3 days, and decreased at 7 days after ICH. These authors also reported that apoptosis was positively correlated with malondialdehyde and free radical content in brain tissue, suggesting that increased oxidative stress injury after ICH may lead to brain cell apoptosis (72). Moreover, intracellular ROS and microglia apoptosis were significantly attenuated by the ROS inhibitor N-acetyl-l-cysteine, probably *via* a thioredoxin interacting protein pathway (65). Thus, inhibition of ROS/thioredoxin interacting protein pathway may ameliorate microglial apoptosis.

OS triggers apoptosis through a number of mechanisms. For example, ROS and oxidation of mitochondrial proteins cause mitochondrial permeability transition pore to open, and cytochrome C is then released to activate cell death intrinsic pathways (70, 73). As well, mitochondrial membranes are destroyed by peroxidated lipids, resulting in the outflow of Ca^2+^ and loss of cell membrane function leading to mitochondrial permeability transition pore formation (70). Moreover, cellular viability is decreased by the 4-hydroxy-trans-2-nonenal-derived OS, which is mainly related to caspase independent apoptosis (74).

#### 4.3.2 OS Results in Brain Cell Necrosis After ICH

Necrosis can be induced by a variety of chemical or biological insult. Necrosis is characterized by swelling of the organelles, rupturing of membranes, and release of cell contents.

Evidence suggest that excessive superoxide generated by nicotinamide adenine dinucleotide phosphate oxidase in cells leads to the switch from apoptosis to necrosis (75). Conversely, administration of nicotinamide adenine dinucleotide decreases OS induced by H_2_O_2_ and protects cells from necrosis (76). Recent data show that inhibiting mitochondrial dysfunction by AdipoRon increased ATP levels, lowered ROS, and reduced neuronal necrosis and the apoptotic rate at 72 hours after ICH in mice (77). These results suggest that changes in intracellular redox status influence the process of cell necrosis. Mechanisms of cell necrosis induced by OS after ICH includes: 1) Mechanical compression of the hematoma on the adjacent tissue and activation of NMDAR by excessive glutamate. Both can result in an influx of calcium, which then causes the depolarization of mitochondrial membrane and the activation of the mitochondrial permeability transition pore, resulting in release of ROS and necrosis (78); and 2) Activated microglia/macrophages and infiltrating neutrophils following ICH can release proteases and oxidants. Moveover, they can also directly induce cell necrosis by attacking complement-bound cells (79).

#### 4.3.3 OS Causes Necroptosis After ICH

Necroptosis is a receptor-interacting protein and mixed lineage kinase domain-like protein mediated programmed necrosis that can be induced by ROS. The ROS scavenger, butylated hydroxyanisole, lowered the number of necroptotic cells; corresponding, necrostatin-1 significantly decreased ROS accumulation induced by hemin *via* inhibiting receptor-interacting protein 1 in HT-22 cells (80). These results suggest that antioxidants ameliorate heme-induced necroptosis (76). There is considerable evidence that ROS can drive necroptosis through caspase independent pathways (81). However, the mechanisms of OS in inducing necroptosis still need be further explored.

#### 4.3.4 OS Activates Autophagy After ICH

Autophagy is a lysosome-mediated degradation mechanism of cellular contents. Moderate autophagy promotes cell survival, while excessive autophagy induces cell death (82). A recent study revealed that ICH involves autophagy and iron plays an important role in this process (83). Consistent with this result, another study reported that autophagy appeared at 6 hours after ICH, the autophagic flux was damaged at 3 days, and returned to normal at 7 days (84). Interestingly, OS is a key mediator to induce autophagy after ICH (71). The mechanisms of OS-mediated autophagy after ICH primarily include three aspects. First, OS promotes autophagy up-regulation to clear dysfunctional mitochondria, destroyed intracellular proteins and other damaged organelles, maintaining intracellular homeostasis (85). Second, OS activates IKKα/NF-κB/I-κB kinase β-mediated pro-inflammatory signaling pathways through autophagy. At the same time, the accumulation of large amounts of ROS in mitochondria and cytoplasm can cause OS damage and induce autophagy. Third, the molecular network of ROS damage, including toll-like receptor-4, PI3K-Akt-mTOR, and AMPK, is closely related to the activation of autophagy-related genes (85).

#### 4.3.5 Os Leads to Ferroptosis After ICH

Ferroptosis is an iron-dependent lipid peroxidation injury of cell. It is characterized by excessive involvement of antioxidant enzymes and the lethal peroxidation of lipid bilayer membrane. Ferroptosis is elicited in ICH and its inhibition is beneficial *in vivo* and *in vitro* ICH conditions (40). The antioxidant enzyme glutathione peroxidase 4 may alleviate secondary brain injury following ICH by inhibiting ferroptosis (86). OS is involved in the process of ferroptosis. Moreover, increased lipoxygenase activity leads to sustained oxidation of phosphatidyl ethanol and arachidonic acid, which is an indispensable part of ferroptosis (81). Administration of ferrostatin-1 decreased lipid oxidation products, and inhibited gene expression of cyclooxygenase-2 and prostaglandin-endoperoxide synthase-2 (42). Therefore, the imbalance of OS due to intracellular iron overload and depletion of antioxidant enzyme may be an important factor of ferroptosis after ICH.

### 4.4 OS Promotes BBB Disruption After ICH

The BBB is an important part of the neurovascular unit, which strictly regulates the exchange of molecules and ions between brain tissue and peripheral blood circulation (87). Disruption of BBB and the formation of brain edema contribute to the fatality of ICH patients (88). The entry into the brain of blood components including thrombin, fibrin, and erythrocyte components initiate inflammation, OS, and cytotoxic edema, which leads to increased permeability of the BBB. ROS produced by iron metabolism can disrupt the BBB through direct degradation of vascular endothelium and activation of downstream signaling pathways (89). Moreover, hemoglobin-induced OS can activate matrix metalloproteinase, leading to endothelial cell apoptosis and BBB dysfunction (27). Unconjugated bilirubin, a source of ROS after ICH, can also induce brain edema and inflammation by disrupting BBB (90). ROS produced by microglia and leukocytes through NOX also propagates BBB damage.

Under the toxic effect of O^2^•−, the tight junctions of endothelium may rearrange or even degrade, leading to the increase of paracellular permeability and the destruction of the integrity of BBB (87). Furthermore, nitric oxide interacts with superoxide to form peroxynitrite, which activates matrix metalloproteinases that disrupt BBB by degrading tight extracellular matrix and junctional proteins (87). As well, oxygen and nitrogen free radicals regulate BBB permeability through destroying endothelial cells and activating associated signaling pathways (90).

## 5 Treatment of ICH With Anti-OS Agents

Because OS is a condition of imbalance between reactive oxygen free radicals and/or deficiency of antioxidant systems, strategies to cope with OS in ICH may include two aspects. The first is through blocking the sources of ROS production or promoting the scavenging of excessive ROS. The second is by interfering with gene expression/protein function of pro-oxidative or anti-oxidative enzymes (**Table 1**). A large number of studies have focused on antioxidant therapy after ICH. Here, we discuss the agents that have been validated in clinical trials and provide the latest information on affecting OS through gene therapy.

**Table 1:** Potential strategies that target OS after intracerebral hemorrhage.

Approaches		Probable mechanism of action or drug targeting	Effects	References
Medications	Deferoxamine	Chelates iron	Reduces iron deposition and release of ROS, inhibits activation of microglia or neutrophil infiltration, and attenuates neuronal death	(91)
	Statin	Downregulation of mevalonate and its derivatives	Reduces TUNEL-positive cells, inducible nitric oxide synthase expression, and myeloperoxidase -positive or OX42-positive cells	(92)
	PPAR-γ	Downregulation of gene tumor necrosis factor, and inducible nitric oxide synthase	Reduces proinflammatory factors expression, extracellular H_2_O_2_ level, and neuronal damage	(93)
	Minocycline	Inhibits metalloproteinases and chelates iron	Prevents neuronal loss and decreases the level of malondialdehyde	(94)
	NXY-059	Scavenges ROS	Decreases neurological impairment, neutrophil infiltrate, and the number of TUNEL-positive cells	(95)
	Edaravone	Scavenges ROS	Alleviates brain edema and decreases the expression of interleukin 1β, Caspase 1 and NF-κB	(96)
Targeted gene therapy	Heme oxygenase-1	The rate-limiting enzyme for heme catabolism and iron production	Heme oxygenase-1 upregulation reduces ROS accumulation and apoptosis rate	(35)
	Heme oxygenase-2		Heme oxygenase-2 gene deletion alleviates the level of carbonylation and malondialdehyde, reduces ROS and cell injury	(33, 97)
	Nrf2	Promotes expression of a broad range of antioxidants	Nrf2 knockout aggravates neurological injury, increases hematoma volume, cytochrome C release, and DNA oxidative injury	(98)

### 5.1 Strategy for ROS Production and Scavenging

#### 5.1.1 Deferoxamine

Iron released by hemoglobin, the degradation product of erythrocytes within hematoma, has been implicated in apoptosis, OS, inflammation, and autophagy, which contribute to secondary brain injury after ICH (99). Thus, targeting iron is a promising strategy against OS in ICH.

Deferoxamine is an iron chelating agent that crosses the BBB to reach high therapeutic concentrations in brain tissue (100). It can prevent iron from entering the Haber–Weiss reaction to produce hydroxyl radicals by forming stable compounds with chelated ferric iron and hemosiderin (91). Moreover, deferoxamine regulates the expression of iron metabolism regulation genes, the activity of binding proteins, and it promotes the gene expression of antioxidant enzyme that inhibit apoptosis. In addition, deferoxamine down-regulates the expression of prolyl 4-hydroxylase and c-Jun N-terminus kinase genes, while it elevates heme oxygenase-1 gene expression after ICH (101). More importantly, deferoxamine has both anti-phagocytic and anti-inflammatory effects (102). Therefore, deferoxamine may be a promising therapy for ICH. Systemic administration of deferoxamine (200 mg/kg, intraperitoneal) lowered ROS, reduced iron deposition, attenuated the activity of microglia and infiltrated neutrophils, and decreased neuronal death in a collagenase-induced ICH model (91). More recently, a clinical trial has confirmed the safety of deferoxamine in ICH patients (103). A *post hoc* analysis of the intracerebral hemorrhage deferoxamine trial (iDEF Ttrial) reported that deferoxamine treated patients with moderate hematoma volume (10-30 mL) achieved favorable outcomes (104).

#### 5.1.2 Statins

Statins, which are classical cholesterol-lowering agents, have pleotropic effects including anti-inflammatory, antioxidative, and neuroprotective outcomes (105). Statin-mediated neuroprotection may stem from several mechanisms, including reduction of ROS production and down-regulation of mevalonate and its derivatives; statins also target the main signaling pathways of cell proliferation, adhesion, migration and cytokine secretion (106). In rats with cecal ligation and puncture, treatment with simvastatin reduced nitric oxide level, increased catalase activity, and normalized reduced/oxidized glutathione (GSH/GSSG) ratio in the brain (107). Atorvastatin decreased TUNEL-positive cells, inducible nitric oxide synthase expression, and myeloperoxidase-positive or OX42-positive cells in the perihematomal regions in ICH in a dose-dependent manner (92). However, another group observed that prior use of statins before ICH did not impact neurological outcomes (108). A clinical trial found that application of statins before onset of ICH reduced the volume of brain edema around hematoma (109) and 30-day mortality (110). These contrary results may be due to species differences or the mode of administration of statin. A phase III trial (Statins In Intracerebral Hemorrhage [SATURN]) is now underway to determine the benefits of statin by continual or discontinuous administration; and whether the individual’s apolipoprotein-E genotype may affect the decision to continue/discontinue statins. This study should provide new insights and guidelines to the use of statin in ICH patients.

#### 5.1.3 Peroxisome Proliferator-Activated Receptor γ Agonists

PPAR-γ is a ligand-activated nuclear transcription factor which plays an important role in augmenting the phagocytosis activity of microglia/macrophage, and regulating oxidative stress and inflammation. Rosiglitazone, a PPAR-γ agonist, upregulated the CD36 phagocytic receptor in phagocytes, enhanced the ability of phagocytes to engulf erythrocytes, promoted hematoma absorption and reduced ICH-induced neurological defects in a mouse model of ICH (111). Consistent with this, another study revealed that PPAR-γ agonists remarkablely enhanced the expression of PPAR-γ targeted genes (CD36 and catalase); they inhibit the transcription of pro-inflammatory factors, reduce extracellular H_2_O_2_ level, and promote neurological function recovery (93). Based on these encouraging preclinical research results, a clinical trial was conducted to test the safety of pioglitazone in the absorption of hematoma after ICH. However, although the study is completed (ClinicalTrials.gov Identifier: NCT00827892), the results have not appeared in the published literature.

#### 5.1.4 Minocycline

Minocycline, a tetracycline derivative, is FDA-approved for bacterial infection and acne vulgaris. It can cross the BBB, and significantly reduce the infiltration of neutrophils and macrophages; it decreases the activation of microglia and ameliorates irreversible damage of cells (112, 113). Minocycline is also a potent matrix metalloproteinase inhibitor; it is an iron chelator and has anti-inflammatory properties (114). In cortical cell cultures treated with ferrous sulfate, minocycline prevented neuronal loss and decreased the level of malondialdehyde (94), which corroborated the anti-OS property of minocycline. Recently, it was shown to significantly improve neurobehavioral outcomes in ICH (115). Moreover, a small clinical trial showed that minocycline at 400 mg per day achieved reasonable serum concentrations in ICH patients (116). A review of the literature has proposed that a high dose twice daily IV minocycline, initiated promptly after ICH with hematomal evacuation, is a reasonable strategy to pursue in a treatment trial in ICH to improve its prognosis (117).

#### 5.1.5 Disodium 4-[(Tert-Butylimino) Methyl] Benzene-1,3-Disulfonate N-Oxide

NXY-059 is a free radical scavenger developed for use in acute ischemic stroke. In a collagenase induced ICH model, NXY-059 treatment significantly inhibited the infiltration of neutrophils, reduced TUNEL-positive cells around the hematoma, and improved the neurological dysfunction of rats 48 hours after ICH (95). A clinical trial was conducted in 2004, and confirmed that NXY-059 was safe to begin within 6 hours of ICH and wass well tolerated with no serious adverse effects; however, there were no obviously differences in neurological deficit scores and disability rates at 3 months after ICH compared with the placebo-treated patients (118). Therefore, the translation of NXY-059 from bench to clinic needs to be further investigated.

#### 5.1.6 Edaravone

Edaravone is a ROS scavenger widely used in ischemic stroke within 24 hours of onset. In ICH animal model, edaravone significantly alleviated brain edema, inhibited the activation of caspase, and decreased the levels of interleukin 1β and NF-κB (96). Moreover, edaravone improved behavioral recovery scores, ameliorated cerebral edema and BBB permeability, decreased interleukin 1β and tumor necrosis factor levels, and ameliorated neuronal apoptosis (119). However, there is no clinical evidence of the effectiveness of edaravone in ICH patients.

#### 5.1.7 Remote Ischemic Conditioning

In transient limb ischemia, remote ischemic conditioning confers neuroprotection in part by countering injury-induced ROS generation in a Nrf2-dependent fashion (120, 121). In animals with ICH, remote ischemic conditioning can attenuate brain edema by upregulating heme oxygenase-1, transferrin, and transferrin receptor (122), and promoting hematoma resolution *via* increasing anti-inflammatory macrophage activation (123). In a multicenter, randomized, controlled clinical trial of 40 patients with ICH, treatment with remote ischemic conditioning for seven consecutive days was safe and well tolerated, promoted the rate of hematoma resolution, and reduced perihematomal edema (124). However, functional outcomes at 90 days were not significantly different from those in the control group possibly due to the small sample (124). A phase III randomized clinical trial with 452 participants is currently underway to evaluate the effect of remote ischemic conditioning on the clinical outcomes of patients with ICH (ClinicalTrials.gov Identifier: NCT04657133). The results of this study are eagerly awaited.

### 5.2 Targeted Gene Therapy

#### 5.2.1 Heme Oxygenase

Heme oxygenase degrades heme into biliverdin, carbon monoxide, and iron. Besides, the breakdown of hemoglobin can be partially catalyzed by heme oxygenase (125). As mentioned above, hemoglobin and its degradation products are key mediators of OS after ICH. Thus, targeting heme oxygenase may be of utility for neuroprotection after ICH.

Heme oxygenase-1, a core enzyme in heme and iron-catalyzed metabolism, is highly expressed in microglia and astrocytes, but has low levels in neurons (32). Hemoglobin can induce heme oxygenase-1 upregulation to reduce ROS accumulation and apoptosis (35). Conversely, downregulating the expression of heme oxygenase-1 by siRNA-mediated knockdown of Nrf2 enhanced the toxicity of hemin on astrocytes (126).

Heme oxygenase-2 is mainly expressed by neurons. Deletion of heme oxygenase-2 attenuated OS injury in the whole-blood injected ICH model in mice (125). Moreover, heme oxygenase-2 gene deletion alleviated the level of carbonylation and malondialdehyde induced by hemoglobin injection (33). In addition, heme oxygenase-2 knockout markedly reduced ROS production and cell damage caused by heme or hemoglobin (97).

#### 5.2.2 The Nuclear Factor Erythroid 2-Related Factor 2

Nrf2 is the master regulatory component of antioxidant stress injury. It initiates the expression of various antioxidant enzymes such as superoxide dismutase, glutathione peroxidase-1, heme oxygenase-1, glutathione transferases, and NAD(P)H quinone oxidoreductase-1 (127). Nrf2 knockout mice showed greater neurological injury, increased hematoma volume, cytochrome C release, and DNA oxidative injury than wild-type ICH mice (98). Similarly, exacerbated neurological injury in Nrf2 knockouts was also reported in the blood injection ICH model (128).

#### 5.2.3 MicroRNAs

miRNAs are a group of small single-stranded RNAs without coding function; they negatively modulate the expression of target genes and proteins (16, 127, 129). OS regulates miRNAs biogenesis by acting on major molecules that influence miRNAs maturation, and modifies miRNAs to alter their integrity, stability and binding activity. Most importantly, multiple miRNAs are involved in antioxidant stress response of cells (127). Accumulating research show that miRNAs are expressed abnormally in perihematomal tissue and blood of patients with ICH (129, 130). However, the effect of different miRNAs on ICH is quite complex and should be discussed within a specific context.

The expression of miR-146a was dramatically reduced in ICH rats (131) and was negatively correlated with OS status in the brain (132). Overexpression of miR-146a inhibited OS injury and inflammation partly through inhibiting the TNF receptor-associated factor 6/NF-κB signaling axis (131). Similarly, overexpressed miR-183-5p protected brain cells and improved neurological deficit function score following ICH *via* blocking heme oxygenase-1 production, indicating heme oxygenase-1 mediated close relationship between Nrf2 and miRNA (129). Moreover, overexpression of microRNA-139 increased Nrf2 expression and decreased NF-κB expression (133). Taken together, miRNAs may exert a protective role by reducing inflammation and OS injury after ICH.

However, the role of miRNA could also be negative. The inhibition of miR-27b, an OS-responsive microRNA, alleviated brain injury and upregulated the level of heme oxygenase-1, superoxide dismutase and Nrf2 after ICH *via* the Nrf2/ARE pathway; dual-luciferase reporter analysis demonstrated that miR-27b acts directly on Nrf2 mRNA (127). Moreover, miR-155 in the parietal cortex and hippocampus was reported to be engaged in neural damage in ICH, and down-regulation of miR-155 expression was beneficial for neurological recovery by reducing the products of OS and enhancing vascular endothelial growth factor (134). In addition, increased miR-130a in serum is closely associated with severe brain edema and poor prognosis after ICH (16).

## 6 Summary and Conclusions of OS Following ICH

OS is a major factor of secondary brain injury following ICH. It can interact and form a vicious cycle with an inflammatory response. Besides, it also disrupts BBB and induces various types of cell death after ICH. Several aspects may contribute to the production of ROS, primarily including mitochondria dysfunction, degradation products of erythrocyte, excitotoxic glutamate, and proinflammatory cells. Both blocking the source of ROS production and eliminating excessive ROS are anti-OS strategies. Moreover, targeting specific OS-related genes is a potential treatment approach for ICH. However, due to the complex processes of ICH where several types of mediators of injury are present, pharmacotherapies targeting OS alone is likely insufficient to ameliorate the pathophysiology of ICH. Therefore, agents targeting OS as well as other mediators of injury would be beneficial for patients with ICH.

## 7 Search Strategy and Selection Criteria

Literature retrieval was performed through PubMed and National Institutes of Health’s Clinical Trials. The keywords “oxidative stress” “ROS” “RNS” “intracerebral hemorrhage” “ICH” and “inflammation” “glutamate” “apoptosis” “necrosis” “necroptosis” “autophagy” “ferroptosis” “blood brain barrier” “antioxidant” “clinical trials” were employed to identify all full text articles in English. Articles were shortlisted based on screening of the title and abstract. The potentially eligible literature on oxidative stress and intracerebral hemorrhage was searched and retrieved. References to related articles were also selected as eligible studies. Review articles that closely related to the topics were included. For clinical trials, the patient was older than 18 years, and for rodent experimental research, the experimental animal was older than 8 weeks. Exclusions include pediatric intracerebral hemorrhage, subarachnoid hemorrhage, traumatic brain hemorrhage, cancerous cerebral hemorrhage, case reports and studies that were published too early (more than 30 years) or not published in English. The final reference list was generated based on the inclusion and exclusion criteria above.

## Author Contributions

All authors listed have made a substantial, direct and intellectual contribution to the work, and approved it for publication.

## Funding

The authors acknowledge operating grant support from the National Natural Science Foundation of China (grants no: 82071331, 81870942, and 81520108011), National Key Research and Development Program of China (grant no: 2018YFC1312200), and from the Canadian Institutes of Health Sciences (VY).

## Conflict of Interest

The authors declare that the research was conducted in the absence of any commercial or financial relationships that could be construed as a potential conflict of interest.

## Publisher’s Note

All claims expressed in this article are solely those of the authors and do not necessarily represent those of their affiliated organizations, or those of the publisher, the editors and the reviewers. Any product that may be evaluated in this article, or claim that may be made by its manufacturer, is not guaranteed or endorsed by the publisher.
